# Reassessing the Role of Copeptin in Emergency Department Admissions for Hypotonic Hyponatremia

**DOI:** 10.1210/clinem/dgaf266

**Published:** 2025-05-03

**Authors:** Alessandro Maria Berton, Emanuele Varaldo, Marco Zavattaro, Stefania Locatelli, Patrizia Ferrera, Emanuele Pivetta, Filippo Gatti, Nunzia Prencipe, Fabio Bioletto, Valentina Gasco, Andrea Silvio Benso, Silvia Grottoli, Paolo Pasquero, Emanuela Arvat, Ezio Ghigo, Enrico Lupia

**Affiliations:** Division of Endocrinology, Diabetology and Metabolism, Department of Medical Sciences, University of Turin, 10126 Turin, Italy; Division of Endocrinology, Diabetology and Metabolism, Department of Medical Sciences, University of Turin, 10126 Turin, Italy; Division of Endocrinology, University Hospital “Maggiore della Carità,” 28100 Novara, Italy; Division of Emergency Medicine and High Dependency Unit, Department of Medical Sciences, University Hospital “Città della Salute e della Scienza di Torino,” 10126 Turin, Italy; Division of Emergency Medicine and High Dependency Unit, Department of Medical Sciences, University Hospital “Città della Salute e della Scienza di Torino,” 10126 Turin, Italy; Division of Emergency Medicine and High Dependency Unit, Department of Medical Sciences, University Hospital “Città della Salute e della Scienza di Torino,” 10126 Turin, Italy; Division of Oncological Endocrinology, Department of Oncology, University Hospital “Città della Salute e della Scienza di Torino,” 10126 Turin, Italy; Division of Endocrinology, Diabetology and Metabolism, Department of Medical Sciences, University of Turin, 10126 Turin, Italy; Division of Endocrinology, Diabetology and Metabolism, Department of Medical Sciences, University of Turin, 10126 Turin, Italy; Division of Endocrinology, Diabetology and Metabolism, Department of Medical Sciences, University of Turin, 10126 Turin, Italy; Division of Endocrinology, Diabetology and Metabolism, Department of Medical Sciences, University of Turin, 10126 Turin, Italy; Division of Endocrinology, Diabetology and Metabolism, Department of Medical Sciences, University of Turin, 10126 Turin, Italy; Division of Internal Medicine 1, Department of Medical Sciences, University Hospital “Città della Salute e della Scienza di Torino,” 10126 Turin, Italy; Division of Oncological Endocrinology, Department of Oncology, University Hospital “Città della Salute e della Scienza di Torino,” 10126 Turin, Italy; Division of Endocrinology, Diabetology and Metabolism, Department of Medical Sciences, University of Turin, 10126 Turin, Italy; Division of Emergency Medicine and High Dependency Unit, Department of Medical Sciences, University Hospital “Città della Salute e della Scienza di Torino,” 10126 Turin, Italy

**Keywords:** urinary sodium, plasma osmolality, extracellular fluid volume, mortality, Charlson Comorbidity Index, NT-proBNP

## Abstract

**Context:**

The role of copeptin in assessing hyponatremic patients at emergency department (ED) admission remains debated.

**Objective:**

This work aimed to assess copeptin's effectiveness in evaluating extracellular fluid (ECF) volume and its predictive value in hyponatremic adults admitted to the medical ED.

**Methods:**

This work comprises a report from the IPSO-URG, a prospective cohort study with recruitment from June 2018 to August 2019 and 6-month follow-up. The setting is a medical ED of a single tertiary center. Patients included a consecutive sample of 123 adults with hyponatremia confirmed by direct and indirect ion-selective electrode assay after glucose correction. Excluding 33 individuals with missing consent or criteria and 6 without hypotonic hyponatremia, 84 patients were analyzed. Data included symptoms, vital signs, ultrasound, medical history, Charlson Comorbidity Index, and pretreatment blood and urine samples. ECF status was reassessed post discharge by 3 endocrinologists, blinded to copeptin results, who classified cases etiologically and resolved disagreements through discussion. In-hospital and 6-month mortality were recorded.

**Results:**

A copeptin-to-urinary sodium (u-Na) ratio less than or equal to 29.5 pmol/mmol increased the likelihood of preserved ECF more than 4-fold (odds ratio 4.28; *P* = .026), outperforming standard u-Na (area under the curve difference 0.177; *P* = .013). Copeptin predicted in-hospital mortality (hazard ratio [HR] 1.005), with greater than 60.1 pmol/L as the optimal cutoff (*P* = .0005). Copeptin (HR 1.005; *P* = .02), N-terminal prohormone of brain natriuretic peptide (HR 1.004; *P* = .031), and comorbidity burden (HR 1.207; *P* = .009) predicted 6-month mortality, with copeptin greater than 13.6 pmol/L indicating a more than 4-fold risk (HR 4.507; *P* = .0001).

**Conclusion:**

Measuring copeptin on ED admission in hypotonic hyponatremia aids diagnosis and mortality prediction. The copeptin/u-Na index more accurately identifies preserved ECF than the standard u-Na cutoff.

Hypotonic hyponatremia is a major electrolyte imbalance in the general population and the most common encountered during hospitalization (1-3).

In acute settings, reduced serum sodium (s-Na) may result from impaired electrolyte-free water clearance due to strong stimulation of hypothalamic arginine vasopressin (AVP) secretion during acute inflammatory states (4). Additionally, in cases of acute-onset or moderate to severe hyponatremia, considerable neurological symptoms may occur due to plasma hypotonicity, which can lead to cerebral edema (5, 6).

All things considered, patients suffering from hypotonic hyponatremia are characterized by a wide variety of signs and symptoms, sometimes serious and leading to access to the emergency department (ED), where a correct diagnostic classification and a timely adequate therapeutic approach are essential (7).

The determination of plasma copeptin (C-terminal portion of pro-AVP, CT-proAVP) in hyponatremia characterization has shown mixed results (8). In fact, on the one hand, copeptin boasts a better correlation with plasma osmolality (p-Osm) than AVP in the presence of lower preanalytical issues, and an accurate automated assay (9, 10). On the other, its determination does not seem capable of simplifying the differential diagnosis of hyponatremia, where persistent secretion of AVP remains the primary mechanism leading to the reduction of electrolyte-free water clearance (11).

Even mild and minimally symptomatic cases of hyponatremia are associated with increased mortality, morbidity, and hospitalization risk (12-16). In particular, chronic reduced s-Na are significant, independent predictor of frailty, irrespective of age (17), a condition associated to an increased risk of disability, hospitalization, postoperative complications and death (18). Moreover, low s-Na levels adversely affect the prognosis of chronic conditions like heart failure (HF) (19-21) and cancer (22, 23).

In this context, copeptin has shown potential in predicting various clinical conditions associated with hypotonic hyponatremia (24-27). It has been recognized for its ability to predict exacerbations of chronic HF, the need for advanced treatments in patients unresponsive to standard therapy (19-21), and the severity of sepsis or pneumonia (28-32). Consistently, some studies suggest that copeptin may have predictive value in hyponatremic patients presenting to the ED, with implications beyond the electrolyte disorder itself (33, 34).

Given the conflicting evidence on copeptin's practical role in hypotonic hyponatremia at ED admission, our primary aims were to evaluate its accuracy in identifying ECF patterns and its predictive value in critically ill patients in our ED.

This is a preliminary report from the IPSO-URG study (ClinicalTrials.gov ID: NCT04402190), whose main objectives were to assess physicians’ diagnostic accuracy in identifying hyponatremia subtypes during initial emergency room (ER) evaluation and on ward admission.

## Materials and Methods

Patients admitted to the medical ED of the “Città della Salute e della Scienza di Torino” University Hospital (Turin, Italy) between June 2018 and August 2019 were screened for hyponatremia on point-of-care blood gas analyzer (BGA). Patients were assessed on weekdays, excluding Fridays and Saturdays, between 8:00 and 18:00, to ensure both availability for urinary examination and the possibility of scheduled follow-up in hospitalized patients.

The inclusion criteria were 1) patients of both sexes aged 18 years or older; 2) detection of moderate to severe hyponatremia (ie, s-Na <130 mmol/L) on venous or arterial BGA; 3) confirmation of hyponatremia (ie, s-Na <135 mmol/L) at indirect ion-selective electrode (ISE) method after correction for blood glucose levels; and 4) signature of the informed consent by the patient or, if unable, by a close relative. There were no predefined exclusion criteria.

Clinical evaluation and collection of blood and urine samples were conducted in the ER after detecting hyponatremia via BGA and before initiating treatment. Data collected included vital signs, physical examination, hyponatremia-related symptoms, collapsibility index of the inferior vena cava (IVC), and pleural or peritoneal effusions when available. The IVC collapsibility index was calculated as follows: (maximum IVC diameter—minimum IVC diameter)/maximum IVC diameter (35). Decision criteria for defining volume status based on IVC diameter and collapsibility are shown in Supplementary Table S1 (36).

Drug history was recorded, with particular attention to diuretics and any treatment potentially interfering with the renin-angiotensin-aldosterone system or AVP. Any subsequent therapeutic intervention aimed at correcting natremia was recorded as well.

In the ER a venous sample was collected for the following analyses: s-Na, serum potassium (s-K), urea, blood glucose, creatinine, N-terminal prohormone of brain natriuretic peptide (NT-proBNP), plasma copeptin, and p-Osm. Blood glucose–corrected s-Na was calculated with the Hillier formula (37) [s-Na_mmol/L_ + (glycemia_mg/dL_—100) × 0.024], and the estimated glomerular filtration rate (eGFR) by the Chronic Kidney Disease Epidemiology Collaboration equation (38). A spot urinary sample was collected for urinary sodium (u-Na) and osmolality (u-Osm) and urine physicochemical examination.

ECF status (ie, preserved, reduced, or increased) was determined through a postdischarge reassessment by 3 independent endocrinologists, who were experts in salt and water disorders, based on vital signs, clinical evaluation, biochemical data, and bedside ultrasonography, including the IVC collapsibility index when available. They were not involved in the patient's management or aware of copeptin results, but they reviewed all other medical records, including the ER physician's opinion and treatment response. The same endocrinologists classified all the cases according to the classification previously proposed by Kumar and Berl (39) (ie, primary polydipsia, renal losses, euvolemic, extrarenal losses, hypervolemia). In case of disagreement, the experts proceeded to a discussion and comparison, to produce a shared opinion.

The Charlson Comorbidity Index (CCI), which predicts 10-year survival in patients with multiple comorbidities, was calculated for each case (40). The date and cause of death were collected through retrospective consultation of data from the National Institute of Statistics.

The study was approved by the local ethics committee (cod. 0026347) and was in accordance with the principles of the Declaration of Helsinki. All participants or, if unable, a close relative gave their informed consent to the processing of their data.

### Laboratory Analysis

Samples for point-of-care BGA were collected into heparinized syringes and analyzed on the GEM Premier 3500 (Instrumentation Laboratory SpA), using a potentiometric direct ISE. The s-Na measurement range was 100 to 200 mmol/L with a resolution of 1 mmol/L. Precision coefficient of variation (CV) was 0.46% to 0.91%, intraseries CV 0.32% to 0.55%, interday CV 0.47% to 1.04%, and total CV 0.7% to 0.86%. The precision calibration validation product interday CV was 0.44% to 0.46% and the total CV 0.55% to 0.56%.

For s-Na measurement, blood samples were collected in Li-heparin tubes, centrifuged, and analyzed on the Cobas 8000 ISE (Roche Diagnostic). The limit of detection (LOD) was 80 mmol/L, with intra-assay and interassay CVs both less than 2%.

Plasma samples for p-Osm evaluation were collected in Li-heparin tubes and analyzed within 1 hour using an automatic osmometer (Osmo Station OM-6050, ARKRAY Global) based on freezing point depression. The LOD was 0 mOsm/kg with an intra-assay CV of less than 1%.

NT-proBNP was measured from blood collected in EDTA tubes, processed on the Cobas e602 automated platform (Roche Diagnostics), including centrifugation and a sandwich immunoassay (Elecsys proBNP II) with electrochemiluminescence detection. The LOD was 5 pg/mL (0.6 pmol/L), with a dynamic range of 5 to 35 000 pg/mL and intra-assay and interassay CVs of less than 5%.

For copeptin, blood was collected in EDTA tubes, centrifuged, and analyzed using the B.R.A.H.M.S. KRYPTOR compact PLUS (Thermo Fisher Scientific) with TRACE (Time-Resolved Amplified Cryptate Emission) technique. The LOD was 0.9 pmol/L, while intra-assay and interassay CVs were less than 7% and less than 12%.

All other routine laboratory tests on serum, plasma, and urine samples were performed using automated biochemical analyses at the central laboratory of our University Hospital.

### Statistical Analysis

Sample size was not calculated for this proof-of-concept study. Normally and nonnormally distributed variables were reported as mean ± SD or median and interquartile range (IQR), respectively, while categorical data were presented as counts and percentages. Normality was tested using the Shapiro-Wilk test. Comparisons between groups were made using the independent *t* test, Mann-Whitney test, Wilcoxon signed-rank test, and Kruskal-Wallis test as appropriate. The chi-square test and Fisher exact test assessed associations between binary variables, and Spearman rank test evaluated correlations between continuous variables.

Univariate and multivariable logistic regression models were calculated to define the association between the different variables and to assess their accuracy in predicting the outcome of interest. The Kaplan-Meier method was used to compare survival curves between groups. Receiver operating characteristic (ROC) analysis identified cutoff values with maximum sensitivity (Se) and specificity (Sp) based on the Youden index. Pairwise comparison of ROC curves tested the statistical significance of differences between areas under curves (AUCs) for the same cases. The multivariable Cox regression model assessed the effect of variables on predicting outcomes.

A *P* value less than .05 was considered statistically significant. Statistical analysis was performed using MedCalc (Statistical Software version 20.007, MedCalc Software Ltd).

## Results

**Figure 1.**
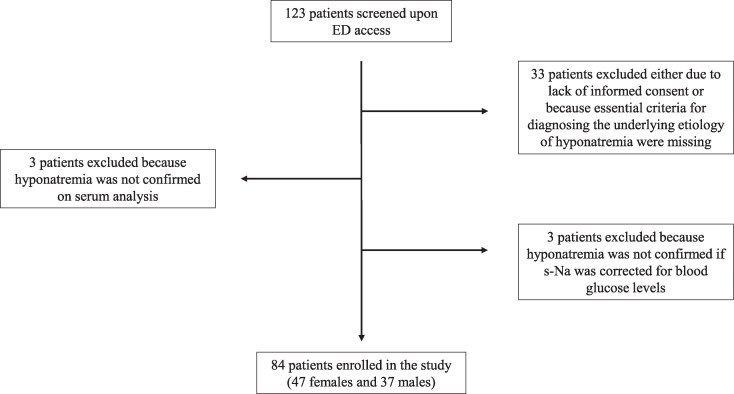
Recruitment process flowchart.

As reported in the recruitment flowchart (Fig. 1), 123 patients were evaluated on admission to the ER. Among these, 33 individuals were excluded either due to the absence of informed consent, or because missing essential criteria for differential diagnosis. In 3 cases hyponatremia was not confirmed by indirect ISE assay, while in another 3 cases, it was not confirmed when s-Na was corrected for glucose levels. In the end, 84 patients (47/37 women/men; median age 79 years [range, 71.5-85 years]) were considered for subsequent analyses. Clinical and biochemical characteristics at ER evaluation are shown in Table 1.

**Table 1.** Clinical and biochemical characteristics at emergency room admission both of surviving and nonsurviving hyponatremic patients at 6-month follow-up

Characteristic	Overall (n = 84)	Survivors (n = 54)	Nonsurvivors (n = 30)	*P*
Age, y	79 (71.5-85)	76.5 (70-84)	84.5 (78-88)	.001
Sex, F	47 (62)	34 (63)	13 (43.3)	.084
DM, n	20 (23.8)	9 (16.7)	11 (36.7)	.040
Active malignancy, n	17 (20.2)	6 (11.1)	11 (36.7)	.006
Systolic BP, mm Hg	125 (110-149)	130 (110-150)	110 (100-133)	.025
Diastolic BP, mm Hg	70 (65-80)	75 (70-80)	70 (60-80)	.103
Differential BP, mm Hg	50 (40-65)	52 (40-70)	40 (35-60)	.032
HR, bpm (*^a^*46 patients)	86 ± 17	88 ± 16	79 ± 19	.088
s-Na direct ISE, mmol/L	124 (119-126)	124 (119-126)	123 (118-126)	.465
s-Na indirect ISE, mmol/L	127 (122-130)	127 (123-131)	126 (122-129)	.167
s-Blood glucose, mg/dL	124 (104-143)	120 (102-137)	135 (119-166)	.009
Corrected s-Na, mmol/L	128 (123-131)	128 (123-131)	127 (123-130)	.418
Severe hyponatremia, n	29 (34.5)	18 (33.3)	11 (36.7)	.760
s-K mmol/L	4.3 (3.6-4.9)	4.2 (3.5-4.6)	4.8 (4.2-5.5)	.003
p-Osm, mOsm/kg	265.5 ± 16.5	263.4 ± 16.0	269.2 ± 17.1	.129
s-Creatinine, mg/dL	0.97 (0.74-1.8)	0.9 (0.73-1.64)	1.4 (0.82-2.09)	.059
pH	7.42 (7.38-7.46)	7.42 (7.38-7.46)	7.41 (7.37-7.48)	.791
eGFR, mL/min/1.73 m^2^	67 (30.5-83.5)	75.8 (39-87)	47 (28-67)	.017
Urea, mg/dL (74 patients)	37.5 (29-80)	36 (25-51.5)	75 (32.75-110.75)	.029
p-copeptin, pmol/L	20.85 (8.9-53.6]	12.5 (6.7-28.3)	54.3 (22-81)	<.0001
p-NT-proBNP, pg/mL (76 patients)	2006.5 (454.5-6871.5]	1120 (335.8-3581.8)	5789 (1304.8-15882.8)	.001
Altered ECF, n	56 (66.7)	32 (59.3)	24 (80)	.055
u-Na, mmol/L (72 patients)	40.5 (20-73)	43 (18.3-69)	38 (20-78.5)	.786
Copeptin/u-Na, pmol/mmol × 100 (72 patients)	52.2 (16.45-208.45)	33.6 (10.73-102.63)	160.7 (49.45-474.85)	.0004
u-Osm, mOsm/kg (74 patients)	350 (263-450)	343 (232.8-459.3)	378 (331.5-429.8)	.090
Charlson Comorbidity Index	5 (4-8)	5 (3-6)	8 (6-8)	<.0001

Data are expressed as mean ± SD or median and interquartile range (IQR) or n (%).

Abbreviations: Altered ECF, reduced or increased ECF; BP, blood pressure; bpm, beats per minute; DM, diabetes mellitus; ECF, extracellular fluid; eGFR, estimated glomerular filtration rate; F, female; HR, heart rate; ISE, ion-selective electrode; NT-proBNP, N-terminal prohormone of brain natriuretic peptide; p-, plasma; p-Osm, plasma osmolality; s-, serum; s-K, serum potassium; s-Na, serum sodium; u-Na, urinary sodium; u-Osm, urine osmolality.

^*a*^HR reported only for patients not on β-blocker treatment.

Natremia measured by BGA was significantly lower than s-Na levels measured by indirect ISE assay (124 [119-126] vs 127 [122-130] mmol/L; *P* < .0001) with an average difference equal to 3 mmol/L. At the time of presentation to the ER, 29 of 84 patients (34.5%) suffered from severe hyponatremia (defined as s-Na corrected for blood glucose <125 mmol/L at indirect ISE) and 44 (52.4%) exhibited some degree of neurological symptoms likely related to plasma hypotonicity. In the ER, a trend was recorded toward an association between the severity of symptoms and the degree of hyponatremia (*P* = .050 for the trend; Supplementary Table S2) (36). A statistically significant association was also found between the class of diuretics administrated at home and the degree of hyponatremia, the association between the epithelial sodium channel inhibitor (ENaC-i) and hydrochlorothiazide being more frequent in severe cases (*P* = .038), and between loop diuretics and mineralocorticoid receptor antagonists in nonsevere ones (*P* = .031; Supplementary Table S3) (36). No patients were receiving sodium-glucose cotransporter type 2 inhibitors (SGLT2-i). IVC collapsibility index recorded during point-of-care ultrasound was available for 34 of 84 patients (40%).

Based on the comprehensive postdischarge reassessment of 84 hyponatremic cases, 28 patients (33.3%) had a reduced ECF, 28 a preserved ECF, and finally, 28 an increased one (Table 2). The 3 blinded experts disagreed in 8 out of 84 cases (9.5%) regarding the precise ECF classification. However, they reached a consensus after discussion, without substantial challenges.

**Table 2.** Clinical and biochemical characteristics at emergency room admission of hyponatremic patients based on extracellular fluid volume estimation

Parameters	Decreased ECF (n = 28)	Preserved ECF (n = 28)	Increased ECF (n = 28)	*P*
Age, y	78 (73-86)	77.5 (69-84.5)	81.5 (74-85)	.431
Sex, F	19 (67.9)	18 (64.3)	10 (35.7)	.029*^b^*
DM, n	7 (25)	2 (7.1)	11 (39.3)	.018
Active malignancy, n	5 (17.9)	3 (10.7)	9 (32.1)	.127
Systolic BP, mm Hg	120 (100-130)	148 (130-155)	113 (110-130)	<.001
Diastolic BP, mm Hg	70 (61-80)	80 (70-90)	70 (60-80)	.004
Differential BP, mm Hg	45 (35-50)	60 (43-73)	45 (40-63)	.009
HR, bpm (46 patients*^a^*)	87 ± 17	85 ± 21	85 ± 12	.963
pH	7.43 (7.38-7.49)	7.43 (7.41-7.46)	7.38 (7.34-7.48)	.154
s-Na direct ISE, mmol/L	123.5 (117-126.5)	123 (119-125)	124 (121-126)	.584
s-Na indirect ISE, mmol/L	127 (118-131)	126 (122-128)	128 (123-131)	.405
s-Blood glucose, mg/dL	125 (108-141)	122 (104-137)	129 (103-147)	.784
Corrected s-Na, mmol/L	127.5 (118-131)	127.5 (122.5-129)	128 (124-131.5)	.405
Severe hyponatremia, n	11 (39.3)	10 (35.7)	8 (28.6)	.692
s-K mmol/L	4.3 (3.5-4.9)	4.2 (3.6-4.5)	4.7 (4.2-5.5)	.052
p-Osm, mOsm/kg	265.2 ± 18.1	259 ± 14.9	272.3 ± 14.1	.009*^c^*
s-creatinine, mg/dL	1.27 (0.79-1.88)	0.77 (0.61-1.95)	1.32 (0.97-2.39)	.0003*^d^*
eGFR, mL/min/1.73 m^2^	46.5 (29-73.5)	82.4 (71-93.2)	47 (20-73.8)	.0003*^d^*
Urea, mg/dL (74 patients)	43 (29-110)	32.5 (25-40)	57 (32.75-86)	.018*^d^*
p-Copeptin, pmol/L	23.65 (8.2-63.1)	12.6 (6.75-29.25)	29.9 (13.45-77.7)	.021*^c^*
p-NT-proBNP, pg/mL (76 patients)	1120 (461.25-5008)	620 (259-2134)	6271 (2093.75-11655.25)	.0004*^b^*
u-Na, mmol/L (72 patients)	30 (12-45)	64.5 (30-122)	37 (13-62)	.009*^d^*
Copeptin/u-Na, pmol/mmol × 100 (72 patients)	126 (40.35-241.4)	17.55 (8.9-50)	96.85 (34.2-231)	.0004*^d^*
u-Osm, mOsm/kg (74 patients)	355 (266.75-461.5)	369 (231-505.75)	343.5 (285.5-380)	.588
Charlson Comorbidity Index	5 (4-8)	4 (3-5)	8 (6-8)	<.0001

Data are expressed as mean ± SD or median and interquartile range (IQR) or n (%).

Abbreviations: BP, blood pressure; bpm, beats per minute; DM, diabetes mellitus; ECF, extracellular fluid; eGFR, estimated glomerular filtration rate; F, female; HR, heart rate; ISE, ion-selective electrode; NT-proBNP, N-terminal prohormone of brain natriuretic peptide; p-plasma; p-Osm, plasma osmolality; s-, serum; s-K, serum potassium; s-Na, serum sodium; u-Na, urinary sodium; u-Osm, urine osmolality.

^*a*^HR reported only for patients not on β-blocker treatment.

^*b*^Increased ECF vs others.

^*c*^Increased vs preserved ECF.

^*d*^Preserved ECF vs others.

Preserved ECF was significantly predicted by higher u-Na levels, even when adjusted for eGFR or diuretic treatment (odds ratio [OR] 1.016; 95% CI, 1.001-1031; *P* = .032). However, the best u-Na cutoff obtained from the ROC analysis (>77 mmol/L, 95% CI, 24-104 mmol/L) was characterized by only modest accuracy in this differential diagnosis as expressed by the AUC (Se 46.1%, Sp 89.1%, AUC 0.717; 95% CI, 0.591-0.834; *P* = .0006). Among other variables significantly associated with preserved ECF, the previously proposed copeptin/u-Na index appeared markedly different among the 3 ECF categories. In the ROC analysis, a copeptin/u-Na cutoff less than or equal to 29.5 pmol/mmol × 100 (95% CI, 17.9-101.1 pmol/mmol × 100, Se 65.4%, Sp 82.6%, AUC 0.782; 95% CI, 0.636-0.877; *P* < .0001) was characterized by the best Youden index, and a copeptin/u-Na ratio at admission to the ER below or equal to this threshold led to a more than quadrupled probability of observing a preserved ECF, even if adjusted for eGFR and diuretic treatment (OR 4.28; 95% CI, 1.191-15.356; *P* = .026).

Although u-Na and copeptin/u-Na index AUCs were not statistically different (*P* = .273), the latter performed significantly better than the most frequently suggested u-Na cutoff of greater than 30 mmol/L (difference between AUCs 0.177; 95% CI, 0.037-0.318; *P* = .013) (Supplementary Fig. S1) (36).

NT-proBNP levels predicted preserved ECF as well (OR 0.984 × 100 pg/mL increase; 95% CI, 0.968-0.999; *P* = .046), though not after adjusting for eGFR. Conversely, increased ECF was predicted by higher NT-proBNP (OR 1.011 × 100 pg/mL increase; 95% CI, 1.003-1.019; *P* = .007), regardless of eGFR or diuretic treatment. ROC analysis showed a cutoff of NT-proBNP greater than 1534 pg/mL (Se 81.5%, Sp 63.3%, AUC 0.760; *P* < .0001) as having the highest Youden index. Notably, NT-proBNP did not predict reduced ECF.

The overall median length of stay was 9 days [range, 6-15 days], while in-hospital mortality was 10.7% (9/84 patients).

The average length of stay of nonsurviving patients was significantly shorter than that of surviving ones (2 [1-6.5] vs 10 [7-15.8] days; *P* = .001).

Copeptin levels at ER admission were significantly higher in nonsurviving patients compared to survivors (median 54.3 [22-81] vs 12.5 [6.7-28.3] pmol/L; *P* < .0001). In contrast, s-Na levels (127 [123-131] mmol/L vs 130 [127-131] mmol/L, survivors vs nonsurvivors) and the CCI (5 [4-8] vs 7 [6-8], survivors vs nonsurvivors) did not show statistically significant differences.

**Figure 2.**
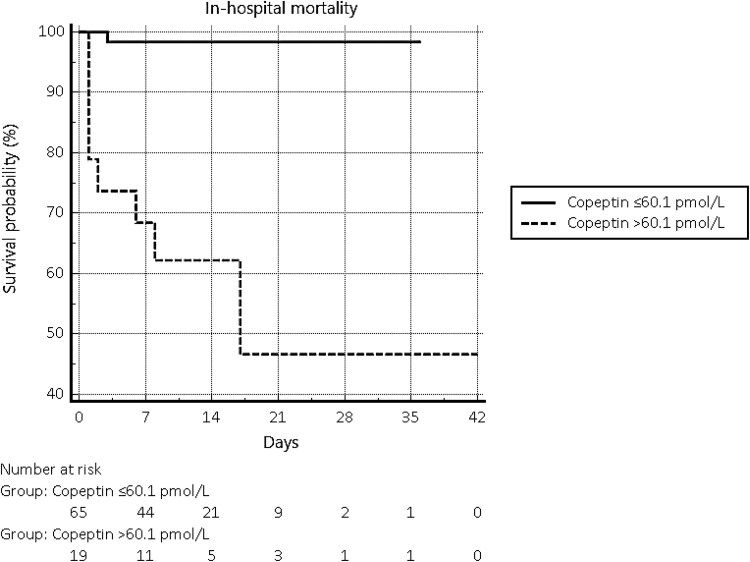
Kaplan-Meier curves describing in-hospital mortality in patients with copeptin levels above or below 60.1 pmol/L on admission to the emergency department (ED).

Copeptin significantly predicted in-hospital mortality (hazard ratio [HR] 1.005; 95% CI, 1.003-1.008; *P* < .0001) and ROC analysis identified copeptin greater than 60.1 pmol/L as the cutoff associated with the best Youden index and likelihood ratios (LRs) (95% CI 58.4-263.2 pmol/L, Se 88.9%, Sp 85.3%, –LR 0.13, +LR 6.06, *P* = .0005). Copeptin values above this threshold recorded at the time of access to the ER led to a marked increase in the risk of death during the hospital stay (HR 59.32; 95% CI, 11.89-295.93; *P* < .0001) (Fig. 2).

Apart from copeptin, both s-K (HR 1.72; 95% CI, 1.02-2.87; *P* = .040) and p-NT-proBNP (HR 1.006 × 100 pg/mL increase; 95% CI, 1.001-1.012; *P* = .022) predicted in-hospital mortality as well; however, given the small group of deceased patients, it was not possible to build a regression model that included more than one independent variable at a time.

Thirty patients (35.7%) died during the 6-month follow-up period. The causes of death for these 30 patients are detailed in Supplementary Table S4 (36). Copeptin was a statistically significant predictor of 6-month mortality, independent of NT-proBNP levels and CCI (copeptin HR 1.005; 95% CI, 1.001-1.009; *P* = .020; NT-proBNP HR 1.004; 95% CI, 1.000-1.007; *P* = .031; CCI HR 1.207; 95% CI, 1.049-1.389; *P* = .009; overall model fit *P* = .0001) (Table 3).

**Table 3.** Multivariable Cox regression model considering copeptin in predicting 6-month patient mortality

Variable	HR (95% CI)	*P*
Copeptin	1.005 (1.001-1.009)	.020
NT-proBNP	1.004 (1.000-1.007)	.031
Charlson Comorbidity Index	1.207 (1.049-1.389)	.009

Abbreviations: HR, hazard ratio; NT-proBNP, N-terminal prohormone of brain natriuretic peptide.

**Figure 3.**
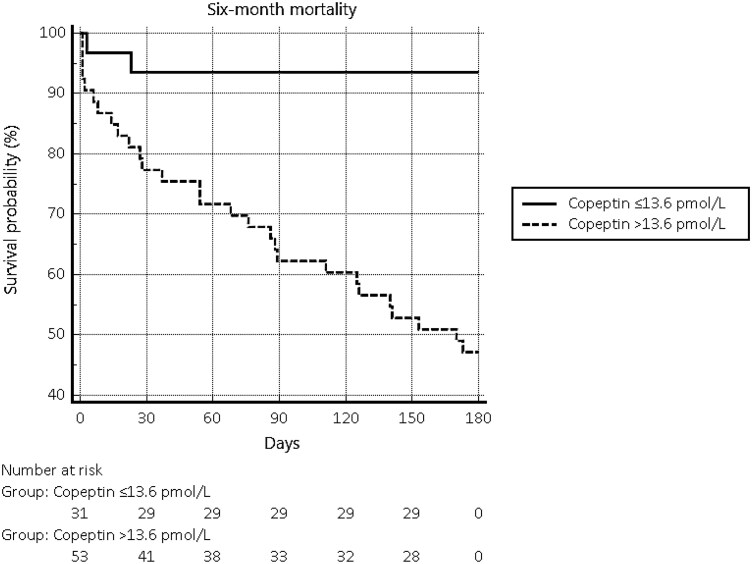
Kaplan-Meier curves describing 6-month mortality in patients with copeptin levels above or below 13.6 pmol/L on admission to the emergency department.

ROC analysis identified copeptin greater than 13.6 pmol/L as the cutoff associated with the best Youden index (95% CI, 8.6-26.3 pmol/L, Se 93.3%, Sp 53.7%, –LR 0.12; *P* < .0001) and values above this threshold led to a more than 4-fold increase in the risk of 6-month mortality (HR 4.507; 95% CI, 2.176-9.334; *P* = .0001) (Fig. 3).

Of note, neither the ECF classification nor the classes of diuretics administrated at home apparently influenced 6-month mortality (Supplementary Tables S5 and S6) (36). Similarly, mortality at last available follow-up was not associated with the etiology of hypotonic hyponatremia, defined according to the Kumar and Berl classification (39) (Supplementary Table S7) (36).

## Discussion

Our study underscores the value of measuring copeptin in patients with hypotonic hyponatremia at ED admission as a strong predictor of 6-month mortality, in conjunction with comorbidity burden. A copeptin level above 13.6 pmol/L at ER entry is highly sensitive and associated with more than a 4-fold increase in the risk of 6-month mortality.

Copeptin is a glycosylated peptide of 39 amino acids, secreted in equimolar amounts with AVP. It likely acts as a chaperone-like molecule for the proper folding of pro-AVP (4). In healthy individuals under normal p-Osm conditions, median copeptin levels are 4.2 pmol/L (range, 1-13.8 pmol/L), with minimal differences between the sexes. Copeptin lacks a significant circadian rhythm and has a longer half-life than AVP, undergoing partial renal clearance due to its low molecular weight (5 kDa). It exhibits low preanalytical variability, and the automated immunofluorescence test requires minimal plasma volume, providing results in approximately 30 minutes (10), making it suitable for managing critical patients in the ER (41).

Copeptin's role is well established in the differential diagnosis of polyuria-polydipsia syndrome; however, its potential applications in assessing ECF status and classifying the etiology of hypotonic hyponatremia have not yet been validated (42-45).

Some previous studies have investigated the predictive role of copeptin in acute patients with hyponatremia. The Biomarkers in Acute Heart Failure (BACH) trial first suggested that copeptin may offer additional information beyond s-Na and NT-proBNP levels, likely serving as an independent predictor both of 90-day mortality and adverse HF outcomes with additive predictive value (33). This conclusion was supported by a subsequent study of an outpatient cohort with class III or IV HF, prospectively evaluated over 2 years for biomarker levels and risk of death or cardiac transplantation. In this study, copeptin was associated with the primary end point, indicating harmful activation of the AVP system even in the absence of overt hyponatremia (46).

In the ED setting, a 2018 prospective multicenter study by Eckart et al (34) involving patients with mild hyponatremia found that both the electrolyte disturbance and high copeptin levels independently increased the risk of all-cause 30-day mortality compared to normonatremia. However, the association between hyponatremia and mortality remained unchanged when copeptin values were added to the regression model, suggesting that the predictive role of hyponatremia was independent of AVP system activation. Unfortunately, this secondary analysis did not document ECF status, urine electrolytes, or concomitant medications (34).

Compared to the latter study, our cohort of hypotonic patients was more severe, with approximately one-third experiencing profound hyponatremia and nearly half presenting with neurological symptoms at ED admission. The median CCI was notably high, particularly among patients with conditions associated with increased ECF and higher copeptin levels. This heightened severity may partly explain the high in-hospital mortality, underscoring the strong short-term predictive value of copeptin levels greater than 60.1 pmol/L for in-hospital death, which appeared to outperform s-Na levels at admission and the CCI. Regardless, our analysis also confirmed that comorbidity burden was the primary predictor of 6-month mortality, alongside, but not superior to, copeptin and NT-proBNP levels at ED presentation. While the predictive value of a unit increase in copeptin is relatively small, values above the 13.6 pmol/L threshold at the ER evaluation led to a more than 4-fold increase in the risk of 6-month mortality. Notably, our cohort did not include normonatremic patients, limiting our ability to estimate the mortality burden specifically due to hyponatremia. However, our findings reinforce that increased AVP release in hypotonic hyponatremia is clinically significant, independent of the degree of electrolyte disturbance.

Nevertheless, it is important to note that our results do not suggest replacing copeptin with other established clinical and biochemical evaluations routinely performed in emergency care, such as s-K or NT-proBNP levels, when clinically appropriate. Rather, the aim of adjusting copeptin's predictive value for these common parameters in our analysis was to confirm its independent contribution.

Supporting the potential pathophysiological role of copeptin in patients’ outcomes, recent literature has highlighted the detrimental effects of chronically elevated copeptin and AVP levels in various clinical conditions. In patients with persistently low effective circulating volume (eg, chronic HF with reduced ejection fraction, advanced cirrhosis), sustained AVP secretion activates vasopressin receptor 1A, promoting myocardial remodeling and fibrosis, leading to increased morbidity and mortality (19, 47). Elevated AVP may also affect albuminuria via vasopressin receptor 2 activation and worsen peripheral insulin resistance in nondiabetic chronic kidney disease patients (48, 49). Moreover, nonosmotically induced AVP secretion, triggered by proinflammatory cytokines, particularly interleukin-6, in infections, can lead to lower s-Na levels and worsen patient outcomes (50). Finally, hyponatremia negatively affects cancer prognosis, with vasopressin receptor 1A activation linked to cell growth, and vasopressin receptor 2 activation showing a proliferative effect in renal carcinoma cells (51).

The second major finding of our research is that, to achieve a reliable clinical assessment of ECF at ER admission, we confirm that u-Na is the most effective single biomarker for identifying preserved extracellular volume in our cohort. However, the copeptin measurement is valuable for calculating the copeptin/u-Na index, which more accurately identifies euvolemic patients than standard u-Na cutoffs. Specifically, a copeptin/u-Na ratio less than or equal to 29.5 pmol/mmol × 100 at ER admission was associated with a more than 4-fold increased likelihood of preserved ECF, even after adjusting for eGFR and diuretic use.

Initial evidence suggested that low copeptin levels (<3 pmol/L) indicate primary polydipsia, supported by very low u-Osm (<200 mOsm/kg) (52), while high levels (>18.9 pmol/L) signal reduced intravascular volume (53). A few years later, Nigro et al (54) conducted the Co-MED study, comparing copeptin's accuracy with other laboratory parameters in diagnosing severe hypotonic hyponatremia based on the Kumar and Berl classification (39). They concluded that copeptin has limited diagnostic utility; only very low values (<3.9 pmol/L) identified primary polydipsia, while high levels (>84 pmol/L) accurately predicted hypovolemia. Of note, despite substantial efforts to define patients’ volume status, only 11% were classified as hypervolemic, 20% as hypovolemic, and the majority (69%) as euvolemic. Additionally, the Co-MED study noted a decline in interest for the copeptin/u-Na ratio, initially proposed by Fenske et al in 2009 (52) to differentiate volume-depleted from normovolemic disorders, as its accuracy for identifying syndrome of inappropriate antidiuresis was similar to that of u-Na levels alone (54).

In our smaller cohort of hypotonic patients, we assessed ECF volume as accurately as possible, including ultrasound evaluation of the ICV when applicable. Our participants were evenly distributed among the 3 categories (reduced, preserved, or increased ECF). The data obtained under these optimal conditions support the ability of the copeptin/u-Na index to accurately identify preserved ECF volume, regardless of residual renal function or ongoing diuretic treatment. Notably, our ROC analysis confirmed a threshold close to that previously proposed by Fenske et al (ie, <30 pmol/mmol × 100) (52), though with a very wide CI. This observation suggests that copeptin/u-Na index values were quite heterogeneous in our cohort, likely primarily driven by the dispersion of u-Na, and that this cutoff should be further validated in larger cohorts. Nevertheless, the copeptin/u-Na index outperformed the widely recommended u-Na cutoff of above 30 mmol/L from international guidelines (5) and expert consensus (6) in our cohort. It is essential to note that, according to these guidelines (5, 6), accurately classifying ECF volume—not the etiology of hypotonic hyponatremia—is crucial for initiating appropriate treatment promptly.

A notable finding in our research was that natremia measured by BGA was slightly but significantly lower—by nearly 3 mmol/L—compared to measurements from a laboratory automated analyzer. Our data are thus consistent with previous works that reported statistically higher results with indirect ISE-based laboratory autoanalyzers compared to BGA (55). ISEs are commonly used for serum electrolyte estimation and include both direct methods (eg, point-of-care BGA) and indirect methods (eg, automated analyzers with preanalytical dilution). BGA is widely used in the ED to provide timely information on electrolytes, glucose levels, acid-base balance, and gas concentrations. Moreover, it is preferred for monitoring s-Na increases during the correction of hypotonic hyponatremia, as the average 90 minutes needed for laboratory results could delay critical decision-making. Nevertheless, indirect ISE methods are generally regarded as more accurate, and the observed overestimation may partly result from sample predilution and decreased serum protein concentrations in critically ill patients. However, experts agree that there are no standard formulas for converting results between direct and indirect ISE methods, so they should not be considered interchangeable (56).

The main strengths of our study include its prospective design and thorough reevaluation of all cases by 3 independent endocrinologists experienced in salt and water disorders, who were blinded to copeptin results but had access to all patient records. Additionally, ECF was assessed using the IVC collapsibility index when available, the CCI was calculated for each patient, and mortality data were obtained directly from the National Institute of Statistics.

Our study does have several limitations. First, the limited cohort consisted of patients recruited only during weekdays and daytime hours, which affects generalizability. Second, some analytes were unavailable for certain patients at the time of ER evaluation. Third, we did not consider additional urinary parameters, such as uric acid fractional excretion (57), urine chloride, and potassium levels (58), which could have been compared for accuracy with the copeptin/u-Na index. Additionally, we acknowledge that copeptin testing is not available in all clinical settings; however, it is becoming more widely accessible, with costs comparable to other common ED tests, such as procalcitonin. Technical issues and personnel requirements have also decreased since its introduction. Finally, copeptin results may be valuable even if available a few hours after ED admission in hyponatremic patients, helping to identify patients who may require intensive care.

In conclusion, our study supports measuring copeptin on ED admission in patients with hypotonic hyponatremia, as it provides valuable predictive and diagnostic information. Copeptin levels reinforce the assessment of mortality risk both for in-hospital and 6-month mortality, in conjunction with comorbidity burden. Additionally, in our well-analyzed and evenly distributed patient cohort, the copeptin/u-Na index demonstrated greater accuracy in identifying preserved ECF compared to the standard u-Na cutoff.

## Data Availability

Some or all data sets generated during and/or analyzed during the current study are not publicly available but are available from the corresponding author on reasonable request.

## Clinical Trial Information

ClinicalTrials.gov registration number NCT04402190 (registered May 22, 2020).

## Abbreviations

AUC: area under the curve
AVP: arginine vasopressin
BGA: blood gas analyzer
CCI: Charlson Comorbidity Index
CV: coefficient of variation
ECF: extracellular fluid
ED: emergency department
eGFR: estimated glomerular filtration rate
ER: emergency room
HF: heart failure
ISE: ion-selective electrode
IVC: inferior vena cava
LOD: limit of detection
LR: likelihood ratio
NT-proBNP: N-terminal prohormone of brain natriuretic peptide
p-Osm: plasma osmolality
ROC: receiver operating characteristic
Se: sensitivity
s-K: serum potassium
s-Na: serum sodium
Sp: specificity
u-Na: urinary sodium
u-Osm: urine osmolality
